# Alterations in the properties of neonatal thalamocortical synapses with time in *in vitro* slices

**DOI:** 10.1371/journal.pone.0171897

**Published:** 2017-02-08

**Authors:** Liliana L. Luz, Stephen P. Currie, Michael I. Daw

**Affiliations:** Centre for Integrative Physiology, George Square, University of Edinburgh, Edinburgh, United Kingdom; University of Exeter, UNITED KINGDOM

## Abstract

New synapses are constantly being generated and lost in the living brain with only a subset of these being stabilized to form an enduring component of neuronal circuitry. The properties of synaptic transmission have primarily been established in a variety of *in vitro* neuronal preparations. It is not clear, however, if newly-formed and persistent synapses contribute to the results of these studies consistently throughout the lifespan of these preparations. In neonatal somatosensory, barrel, cortex we have previously hypothesized that a population of thalamocortical synapses displaying unusually slow kinetics represent newly-formed, default-transient synapses. This clear phenotype would provide an ideal tool to investigate if such newly formed synapses consistently contribute to synaptic transmission throughout a normal experimental protocol. We show that the proportion of synapses recorded *in vitro* displaying slow kinetics decreases with time after brain slice preparation. However, slow synapses persist *in vitro* in the presence of either minocycline, an inhibitor of microglia-mediated synapse elimination, or the TrkB agonist 7,8-dihydroxyflavone a promoter of synapse formation. These findings show that the observed properties of synaptic transmission may systematically change with time *in vitro* in a standard brain slice preparation.

## Introduction

Synaptic communication underlies all brain function and studying the mechanisms behind this communication is vital to understanding how the brain works in both health and disease. Much of our knowledge about synaptic function is based on electrophysiology carried out in a variety of acute *in vitro* preparations. The relevance of these studies relies on synapses in these preparations being representative of synapses *in vivo*. Imaging studies using dendritic spines as a proxy for the presence of synapses have shown that synapses are constantly turned over *in vivo* [1] and have identified populations with very different half-lives [2]. This is particularly apparent in immature animals where the rate of dendritic spine formation is high but the majority of these spines are transient [3]with a narrow window during the first 24hrs in which newly formed spines may become stabilized [4]In contrast, one study showed that, whilst synapses are lost spontaneously, formation of new synapses is limited *in vitro* [5]. This raises the possibility that, if transient and stable synapses truly represent discrete populations with different properties, average synaptic properties may become over-represented by those of persistent synapses with increasing time *in vitro*.

Recently we studied the role of sensory experience in the developmental increase in thalamocortical (TC) connectivity to the neonatal barrel cortex. Experience is often presumed to promote the one-to-one whisker-to-barrel relationship by driving long-term potentiation (LTP) at TC synapses encoding behaviourally-relevant information. It was surprising, therefore, that we found that experience does not alter the strength of individual connections but increases the number of cortical neurons innervated by each TC axon [6]. We hypothesized that there is a constant process of new synapse formation but that these synapses are, by default, transient only being stabilized by experience-driven LTP. This hypothesis is supported by the finding that LTP increases the longevity of newly-formed spines [7] and novel experience in vivo similarly stabilizes a subset of new spines [8]. During this neonatal period two classes of TC synapses can be distinguished by receptor kinetics [9, 10]. In very young (postnatal day (P) 3–7) cortex a large proportion of synapses display slow kinetics but such synapses are almost absent by the end of the TC critical period. Furthermore, slow synapses can be converted to fast synapses by N-methyl-D-aspartate (NMDA)-receptor-dependent LTP [10]. These properties make slow TC synapses a likely candidate for the newly-formed, transient synapses we hypothesized. In this case slow synapses would have a short life-time unless stabilized by LTP. These two easily distinguishable populations of synaptic properties make neonatal TC synapses an ideal candidate to study if there is a disproportionate loss of transient synapses *in vitro*.

Here show that time *in vitro* has a marked impact on the proportion of slow and fast kinetics synapses recorded. Consistent with the idea that transient synapses may be under-represented in older slices there was a decreasing incidence of slow synapses with time after brain slice preparation. Furthermore, slow synapses persist in vitro in the presence of either minocycline, an inhibitor of microglia-mediated synapse elimination, or a promoter of synapse formation, the TrkB agonist 7,8-dihydroxyflavone (DHF).

## Materials and methods

All animal experiments were approved by a University of Edinburgh internal ethics committee and were performed under license by the UK Home Office. 500 μm thick TC slices were prepared from P3 to P7 (P0 is designated as the day of birth) CD1 and C57Bl6jOla (as stated in results), mouse pups as described previously [11, 12]. Briefly, mice were decapitated, the brain removed and placed in an ice-cold partial sucrose solution containing 80 mM NaCl, 2.5 mM KCl, 1.25 mM NaH_2_PO_4_, 25 mM NaHCO_3_, 10 mM glucose, 90 mM sucrose, 4.5 mM MgSO_4_, and 0.5 mM CaCl_2._ The brain was then cut at 50° to the midline and glued to the stage of a vibrating microtome on the cut surface. After cutting, slices were stored at room temperature for at least 1 hr in cutting solution before recording. Slices were transferred to a recording chamber and perfused with an extracellular solution as follows: 130 mM NaCl, 2.5 mM KCl, 1.25 mM NaH_2_PO_4_, 25 mM NaHCO_3_, 10 mM glucose, 1.5 mM MgSO_4_, 2.5 mM CaCl_2_ and 5 μM picrotoxin to block GABA_A_ receptors, thus isolating monosynaptic TC excitatory postsynaptic currents (EPSCs) from the powerful GABA_A_ receptor-mediated feedforward inhibition in barrel cortex [12, 13], and saturated with 95% O_2_/5% CO_2_, pH 7.4, at 33–35°C. For experiments in which slices were incubated in drugs (D-amino-5-phosphonovaleric acid (APV), DHFor minocycline) the drugs were included both in the storage solution immediately after slicing and in the subsequent recording solution. Patch-clamp recordings were made from neurons in layer IV using infrared illumination and differential interference contrast (DIC) optics. Whole-cell recordings were made with patch electrodes (4–7 MΩ) filled with 135 mM Cs methanesulfonate, 8 mM NaCl, 10 mM HEPES, 0.5 mM EGTA, 0.3 mM Na-GTP, and 4 mM Mg-ATP, pH 7.3, 290 mOsm. Thalamocortical EPSCs were evoked at a frequency of 0.2 Hz by electrical stimulation of TC axons by a bipolar stimulating electrode placed in the ventrobasal thalamus. To achieve minimal stimulation conditions stimulus intensity was turned down until no EPSC was seen then increased until the minimum intensity at which an EPSC was observed. Failures were determined by visual inspection. The small amplitude and slow kinetics of slow msEPSCs makes automated detection highly challenging. All kinetics parameters were derived from average EPSCs from all trials excluding failures. 10–90% rise time and fast decay time constant (tau fast) derived from a double exponential fit of the EPSC (both determined in Signal 4, CED) were used to categorize EPSCs (see results). The time of recording was taken as the time at the commencement of individual whole-cell recordings relative to the time at which brain slicing finished. Typically, 1–3 recordings were made from each slice and these recordings were treated separately and, as such n refers an individual recording/cell.

Recordings were made using a Multiclamp 700B (Molecular Devices) were filtered at 4kHz, digitized at 10 kHz and stored on computer using Signal 4.

All p-values were the result of logistic regression analysis (Sigmaplot).

## Results

### Selective loss of slow kinetics msEPSCs in vitro

**Fig 1:**
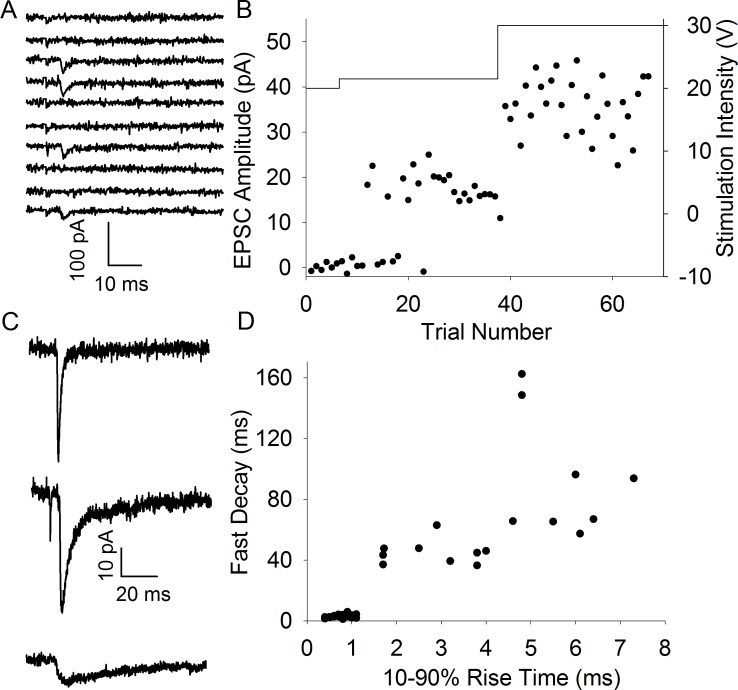
Minimal Stimulation Evokes Thalamocortical EPSCs with Fast and Slow Kinetics. A. Consecutive traces from a representative minimal stimulation experiment showing interleaved EPSCs and failures. B. Amplitude vs trial number plot for experiment shown in A. Solid line shows stimulus intensity (right y axis). C. Representative average traces from individual cells showing fast (top), mixed (middle) and slow (bottom) kinetics. D. Rise time vs fast decay time constant for all minimal stimulation experiments in CD1 mice.

**Fig 2:**
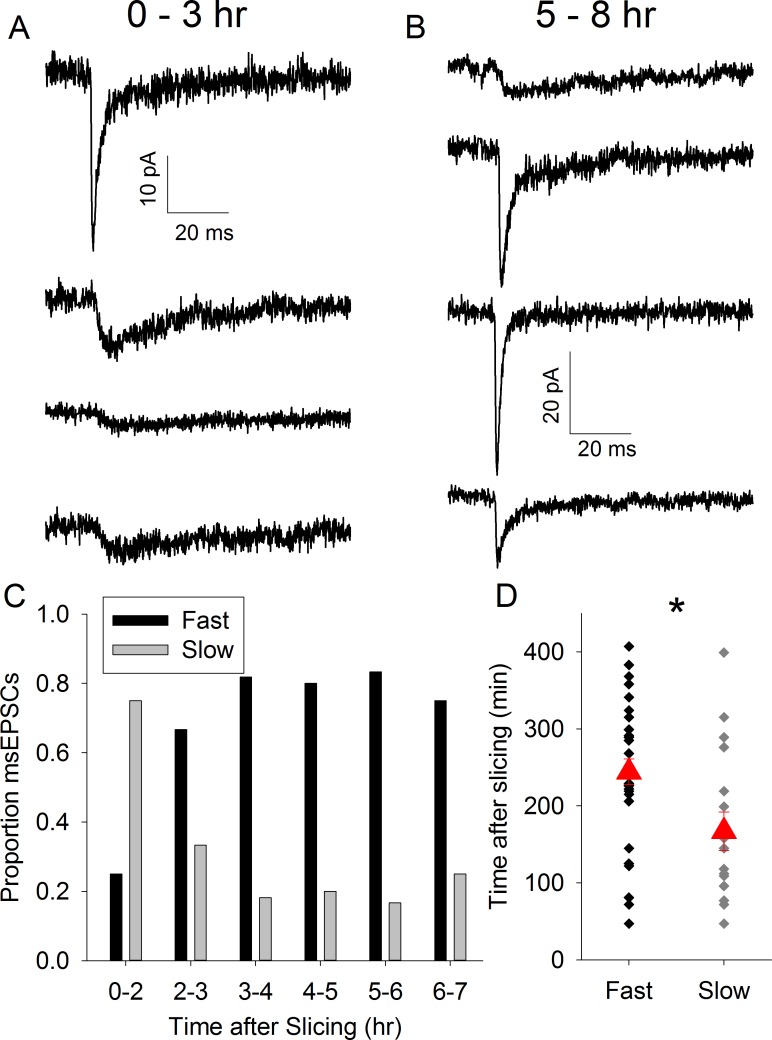
Proportion of msEPSCs Displaying Slow-Only Kinetics Decrease with Time *In Vitro*. A. Average msEPSC traces from representative recordings 0–3 hr after slice preparation. B. Average msEPSC traces from representative recordings 5–8 hr after slice preparation. C. Bar chart showing proportion of of msEPSCs displaying fast/mixed (black) or slow (grey) kinetics in time bins indicated. D. Scatter plot showing time of recording of individual fast/mixed and slow msEPSCs after slice preparation in CD1 mice. Red triangles represent mean ± standard error of the mean (s.e.m.) time of all recordings in group. * represents p = <0.05.

We hypothesized that if there is an absence of synapse formation *in vitro*, the proportion of synapses displaying slow kinetics would decrease as a function of time after brain slice preparation. To test this we initially reanalysed a set of experiments used in a previous study using CD1 mice [6]. In these experiments we evoked TC EPSCs using a minimal stimulation (msEPSCs) protocol to examine the properties of synapses formed by single TC axons (Fig 1A and 1B). EPSCs displayed 3 kinetics profiles: fast, slow and mixed (Fig 1C), the latter representing axons which form separate fast and slow synapses on to the same neuron [9]. The presence of a small slow component in an otherwise fast EPSC can be difficult to identify so we separated the EPSCs in to two groups: fast and mixed combined (subsequently referred to as “fast”) and slow-only. We determined the 10–90% rise time for each msEPSC and fitted the decay with a double exponential. Fig 1D shows that the 2 groups are well-separated using the rise time and the fast decay time constant (Fast: rise time 0.8 ± 0.0 ms, tau fast 2.8 ± 0.2 ms, n = 32. Slow: rise time 4.2 ± 0.4 ms, tau fast 68.3 ± 9.3 ms, n = 17). Next we determined the incidence of slow and fast msEPSCs in one hour time bins relative to time of slice preparation. We found that in the first 2 hours (first two hours pooled due small number of recordings, n = 2, in the first hour) after slicing the majority of msEPSCs display slow kinetics but this proportion rapidly decreases (Fig 2A–2C). In order to avoid the arbitrary nature of time-bins we plotted the time of recording relative to the time at which slice preparation was completed for all recordings and used logistic regression to test for an effect of time on the probability of observing fast or slow EPSCs. We found that the average time of recording slow msEPSCs was substantially earlier than fast (Fast: 244 ± 17 min after slice preparation, n = 32, Slow: 167 ± 25 min earlier than mean time, n = 17, p = 0.015, Fig 2D. Mean time of all recordings: 217 ± 15 min).

### APV does not prevent loss of slow msEPSCs

**Fig 3:**
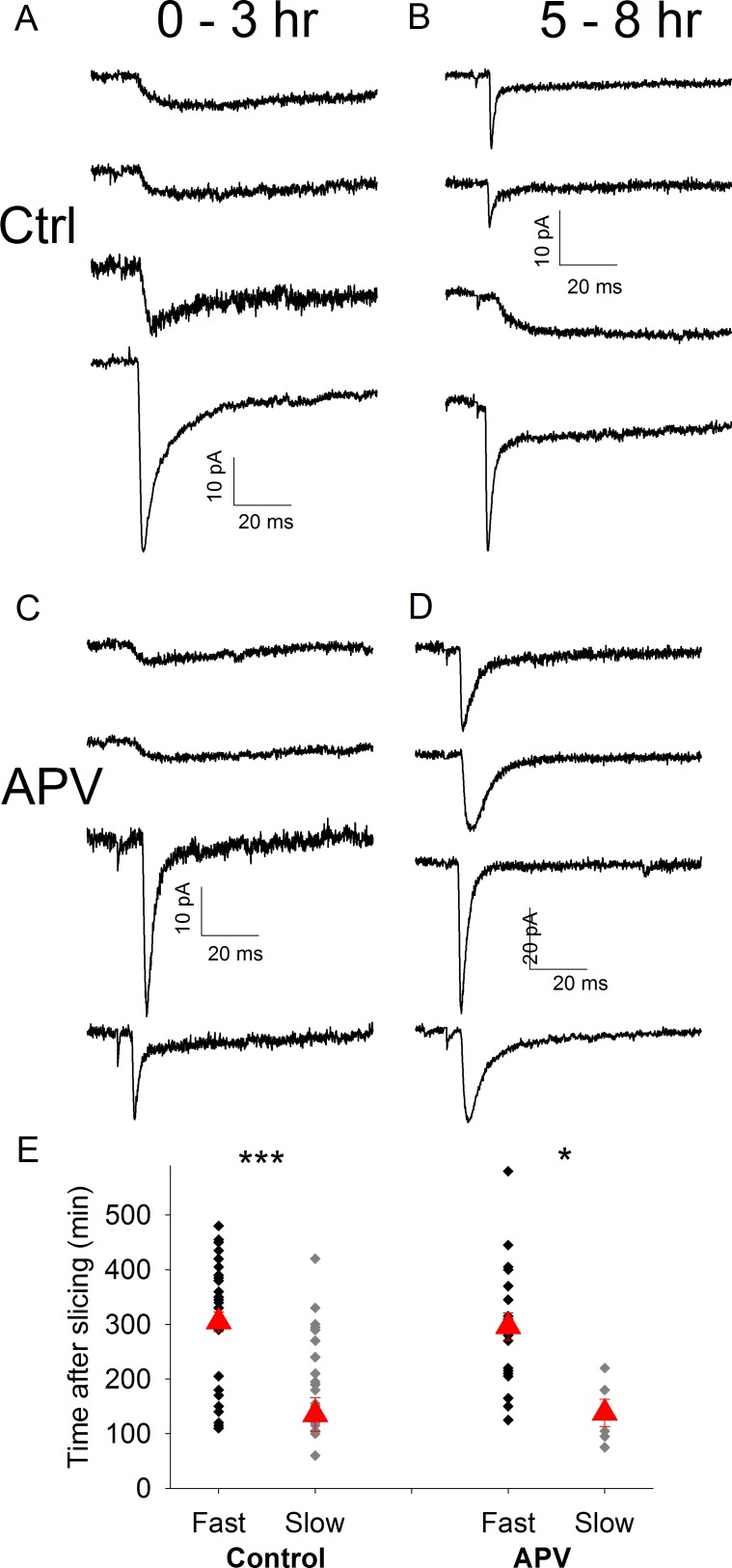
Loss of Slow msEPSCs also occurs in C57/Bl6 Mice and is Not Dependent on NMDA Receptor Activation. A. Average msEPSC traces from representative recordings 0–3hrs after slice preparation in control conditions in C57/Bl6 mice. B. As in A except 5–8 hr after slice preparation. C. Average msEPSC traces from representative recordings 0–3hrs after slice preparation in the presence of 50μM D-APV. D. As in C except 5–8 hr after slice preparation. E. Scatter plot showing time of recording of individual fast/mixed and slow msEPSCs after slice preparation in C57 mice in control conditions and in the presence of 50μM D-APV. Red triangles represent mean ± s.e.m. time of all recordings in group. * & *** represent p = < 0.05 & 0.0001 respectively.

As slow synapses are converted to fast by LTP [14] an alternative explanation for this finding is that the loss of slow synapses represents an LTP-like process in the slice. TC barrel cortex LTP is NMDA receptor-dependent so to prevent spontaneous induction of LTP slices were stored and recorded in a solution containing 50 μM APV. To ensure that the loss of slow synapses also occurs in our current experimental conditions (including the use of C57/Bl6jOla mice) we also performed additional control recordings interleaved with these and all subsequent experiments. These additional control experiments confirmed the original finding: slow msEPSCs were recorded preferentially in experiments with a short time delay after slice preparation (Slow: 195 ± 19 min after slice preparation, n = 23; Fast: 302 ± 18 min after slice preparation, n = 40, p = 0.0009, Fig 3A, 3B and 3E Mean time of all recordings: 263 ± 14 min). Kinetics of slow and fast EPSCs were similar to those in CD1 mice (Slow EPSCs: rise time 14.3 ± 2.1 ms, tau fast 149.5 ± 19.1 ms; Fast EPSCs rise time 0.9 ± 0.1 ms, tau fast 3.5 ± 0.4 ms). In slices incubated in APV we also observed that average recording time of slow synapses was earlier than that of fast synapses confirming that the loss of slow synapses is not a result of LTP induction (Slow: 135 ± 31 min after slice preparation, n = 5; Fast: 296 ± 25 after mean time, n = 21, p = 0.025, Fig 3C–3E. Mean time of all recordings: 261 ± 22 min Slow EPSCs: rise time 9.5 ± 2.0 ms, tau fast 105.0 ± 27.8 ms; Fast EPSCs rise time 0.9 ± 0.1 ms, tau fast 3.3 ± 0.4 ms).

### Inhibition of microglia-mediated synapse elimination prevents loss of slow msEPSCs

**Fig 4:**
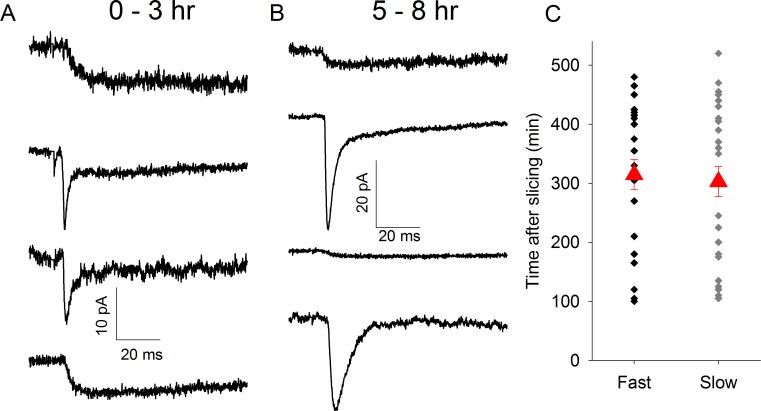
Microglia Inhibitor Minocycline Prevents Loss of Slow Synapses. A. Average msEPSC traces from representative recordings 0–3hrs after slice preparation in the presence of 2μM minocycline. B. As in A except 5–8hr after slice preparation. C Scatter plot showing time of recording of individual fast/mixed and slow msEPSCs after slice preparation in the presence of 2μM minocycline. Red triangles represent mean ± s.e.m. time of all recordings in group.

The lack of effect of APV suggests that rather than being converted to fast synapses slow synapses are lost in *in vitro* brain slices. We questioned whether this synapse loss occurs via normal physiological pathways. A prominent form of synapse elimination involves the engulfment of synaptic terminals by microglia [15]. To test if slow-kinetics synapses are eliminated *in vitro* by microglial engulfment we pre-incubated slices in the tetracycline antibiotic minocycline (2 μM) which has been shown to inhibit microglia-mediated synapse elimination [15]. We found that minocycline completely removed the relationship between time after slice preparation and occurrence of slow msEPSCs (Slow: 303 ± 26 min after slice preparation, n = 30; Fast: 314 ± 26 after slice preparation, n = 25, p = 0.91, Fig 4A–4C. Mean time of all recordings: 308 ± 18 min Slow EPSCs: rise time 16.7 ± 3.8 ms, tau fast 167.9 ± 36.2 ms; Fast EPSCs rise time 1.0 ± 0.1 ms, tau fast 3.3 ± 0.3 ms) consistent with active elimination of slow synapses *in vitro*.

### Activating TrkB receptors prevents loss of slow msEPSCs

**Fig 5:**
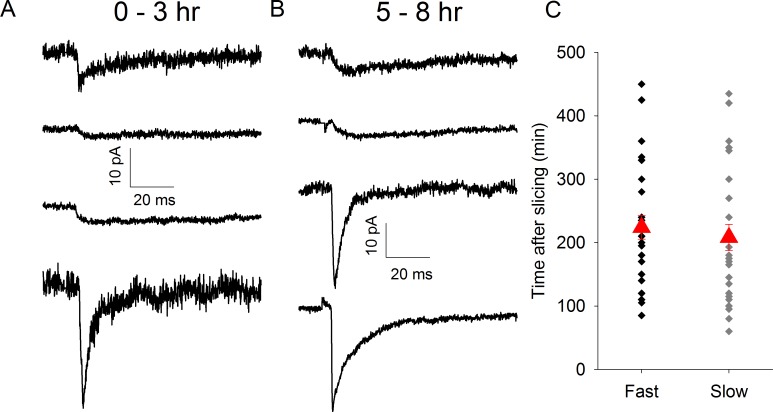
Trk-B Agonist 7,8-dihydroxyflavone Prevents Loss of Slow Synapses. A. Average msEPSC traces from representative recordings 0–3hrs after slice preparation in the presence of 1μM DHF. B. As in A except 5–8hr after slice preparation. C Scatter plot showing time of recording of individual fast/mixed and slow msEPSCs after slice preparation in the presence of 1μM DHF. Red triangles represent mean ± s.e.m. time of all recordings in group.

The loss of axon complexity that accompanies sensory-deprivation induced retardation of TC connectivity *in vivo* [6] suggests that new synapse formation is intrinsically linked with axon growth. We reasoned, therefore, that promoting axon growth *in vitro* would result in *de novo* generation of slow synapses. Brain-derived neurotrophic factor (BDNF) promotes axon growth [16] and, furthermore, BDNF released by microglia and acting via TrkB receptors promotes *de novo* synapse formation [17]. To test if promoting new synapse formation can replace the loss of slow synapses *in vitro* we pre-incubated slices in the TrkB DHF (1μM). In the presence of DHF there was no longer an influence of time *in vitro* on the occurrence of slow and fast msEPSCs (Slow: 208 ± 21 min after slice preparation, n = 28; Fast: 224 ± 19 min after slice preparation, n = 30, p = 0.56, Fig 5A–5C. Mean time of all recordings: 21616 ± 14 min Slow EPSCs: rise time 14.3 ± 2.5 ms, tau fast 171.8 ± 18.8 ms; Fast EPSCs rise time 1.0 ± 0.1 ms, tau fast 3.7 ± 0.4 ms).

## Discussion

Much of our understanding of synaptic function comes from *in vitro* brain slice studies in which experiments are carried out at a varying intervals after preparation of these slices and the data pooled across all these experiments. Here we show that, in a neonatal cortical slice preparation, the proportion of synapses displaying each of two distinct kinetics phenotypes changes dramatically with time after slice preparation. Furthermore, we show that this change in proportion is not due to NMDA receptor-dependent synaptic plasticity but that it is prevented either by DHF, a TrkB receptor agonist or minocycline, an inhibitor of microglia-mediated synapse elimination.

These findings show that time *in vitro* is a significant variable in acute brain slice experiments. In neonatal cortical slices we have found that the proportion of newly-formed synapses displaying slow kinetics decreases with time. Whilst these slow kinetics may be a specific phenotype of neonatal TC synapses dendritic spine imaging studies at other classes of synapses support the concept that newly formed synapses are transient unless stabilized by LTP [4, 7]. It is likely that unidentified properties of these synapses also differ from those of established synapses and that the result of investigations of these synaptic properties may vary with time *in vitro*. The unusual kinetics of the slow EPSCs makes identification of two populations possible at neonatal TC synapses. A similar study would not be possible at other classes of synapse but it seems likely that time-dependent changes are also seen in other brain areas and developmental stages. Indeed it has previously been shown that slicing affects the density of morphologically-identified synapses in mature hippocampus [18, 19]. These studies show that synapses initially disappear upon slicing, seemingly a result of the chilling process, before proliferating to a greater density than that seen *in vivo*. This phenomenon is reduced but not eliminated by the use of a sucrose-based cutting solution similar to that used in our study [18]. This is potentially at odds with finding from our previous *in vitro* studies of the TC system, in slices from animals slightly older than those in this study [6], in which we found a very similar level of connectivity to that seen *in vivo* [20]. The decrease in unitary connectivity observed by le Be et al [5] also argues against a time-dependent increase in connectivity. These varied findings probably suggest that changes *in vitro* are highly dependent on a number of parameters potentially including age, brain area, slicing procedure and solutions and specific synapse class. Nonetheless, the consistent message is that changes in synapse function occur *in vitro* and that these changes must be considered Further, anatomical and electrophysiological changes have been long recognized [21] but are rarely systematically considered. A sensible precaution is simply to routinely assess if time *in vitro* influences findings of brain slice experiments. Such precautions are not unprecedented as it has been demonstrated that the presence of protein synthesis-dependent synaptic plasticity is also affected by time *in vitro* [22] and many researchers in this field now standardize slice incubation time before performing experiments.

Our findings suggest that newly-formed synapses in neonatal cortex display markedly different kinetic properties from stabilized synapses. There are few previous reports of the physiological properties of newly-formed synapses, however, glutamate uncaging-evoked currents from newly formed spines display only very subtle differences to those evoked at persistent spines [23]. The somatosensory L4 neurons studied here do not possess a significant number of dendritic spines until around P9 [24] by which time the vast majority of TC synapses display fast kinetics [10]. This suggests that these slow kinetics may be a specific property of newly-formed dendritic shaft synapses in neonatal cortex. Although the peak current amplitude of slow synapses is small, the long duration of the current results in significant depolarisation for tens of milliseconds [25]. This long depolarisation window may allow induction of the NMDA receptor-dependent LTP that is required for synapse stabilization at an age at which activity levels *in vivo* are relatively low with long periods of silence [26]. This is consistent with the role we propose for these synapses in which axons constantly form synapses on to nearby dendrites but that their fate is determined by an experience-dependent stabilization process [6]. Interestingly the effect of minocycline in preventing the loss of slow synapses suggests that these synapses may undergo microglia-mediated elimination *in vitro*. The effects of minocycline on developing cortex are often attributed not to its microglia action but to inhibition of matrix metalloproteinases (MMPs) especially MMP2 and MMP9 [27, 28]. In this study, however, we incubated slices in 2μM minocycline, a concentration which has been directly shown to inhibit microglia-mediated synapses elimination but is at least 30 times lower than its IC50 for MMP inhibition in any isoforms tested [29, 30]. Furthermore, MMPs have mainly been implicated in long-term potentiation of synapses [31]. The lack of effect of APV we observed argues against inhibition of LTP being responsible for the effect of minocycline.

Our data point to a role for microglia in eliminating non-potentiated synapses in neonatal barrel cortex in common to a similar role they perform in the developing thalamus [15]. A role for microglia in barrel cortex development has previously been demonstrated whereby loss of a key microglial receptor CX3CR1 delays the developmental increase in α-amino-3-hydroxy-5-methyl-4-isoxazolepropionic acid (AMPA):NMDA ratio at TC synapses [32]. If loss of this receptor prevents microglia-mediated synapse elimination this apparent developmental delay in synaptic properties may actually represent an excess number of non-potentiated synapses which have not undergone elimination. Microglial dysfunction during development has also been implicated in autism spectrum disorders [33] and clearly their role in synapse elimination could be linked to the altered patterns of connectivity reported in the brains of autistic patients [34].

Microglia can also support formation of new synapses via release of BDNF which activated neuronal TrkB receptors [17] and BDNF can also promote axon growth which may also contribute to new synapse formation [16]. As such our finding that DHF, a TrkB agonist, prevented the disappearance of slow msEPSCs is consistent with the idea that promoting new synapse formation *in vitro* can compensate for the loss of transient synapses. There is, however, an extremely broad range of downstream consequences of TrKB receptors activation [35] so that a number of other mechanisms could be imagined. For example, BDNF has been shown to stabilize synapses during the development of the neuromuscular junction [36]. If a similar mechanism exists at developing central nervous system synapses, DHF may instead act by preventing the loss of synapses.

In summary we have shown that the proportion of neonatal TC synapses displaying slow kinetics decreases with time in acute brain slices. This has important implications for the interpretation of data from experiments using brain slice preparations. Our data also suggest that slow TC synapse represent newly-formed synapses which are transient by default unless stabilized by LTP.
